# Substance P release from rat dura mater is inversely correlated with CGRP release– experiments using glycerol trinitrate and anti-CGRP antibodies

**DOI:** 10.1186/s10194-025-02050-y

**Published:** 2025-05-16

**Authors:** Mária Dux, Karl Messlinger

**Affiliations:** 1 Department of Physiology, University of Szeged, Dóm Tér 10, Szeged, 6720 Hungary; 2 Institute of Physiology and Pathophysiology, Friedrich-Alexander-Universität Erlangen-Nürnberg, Universitätsstr. 17, Erlangen, D-91054 Germany

**Keywords:** Calcitonin gene-related peptide, Substance P, Cranial dura mater, Anti-CGRP monoclonal antibodies, Neurogenic inflammation, Migraine

## Abstract

**Background:**

The neuropeptides calcitonin gene-related peptide (CGRP) and substance P are important mediators of neurogenic inflammation when they are released from activated primary nociceptive afferents. It is long evident that neuropeptides play an important role in migraine pathophysiology, but the significance of neurogenic inflammation is still debated.

**Methods:**

In an approved hemisected rodent head preparation, we measured CGRP release from the cranial dura mater in parallel with substance P release using animals pre-treated with anti-CGRP antibodies or control solutions.

**Results:**

Apart from the known decrease in CGRP release following antibody treatment, we found a significant inverse correlation of basal and stimulated CGRP versus substance P release across all experiments. The results are discussed in connection with our previously published data.

**Conclusions:**

An increase in CGRP release seems to inhibit substance P release in meningeal structures possibly decreasing substance P-dependent plasma extravasation, which argues against a significant role of neurogenic inflammation in migraine.

## Introduction

Calcitonin gene-related peptide (CGRP) and substance P (SP) are long known neuropeptides present in small peptidergic primary afferent neurons, i.e., nociceptors with C- and Aδ-fibers [1–5], but also in other neural structures including the many areas of the brain [6–11]. It is generally thought that these neuropeptides are stored in dense-core vesicles [12–17]. They can be released upon noxious stimulation from peripheral as well as central endings of the primary afferent neurons [1, 18, 19] and also from cell bodies [20–22] and even from axons within peripheral nerves [23, 24]. Their release depends on a rise in intracellular calcium [23, 25–27], which implies an exocytotic mechanism [28, 29]. CGRP has been found to exist in higher proportions of afferents or at a higher concentration compared to SP. It is generally thought that both peptides co-exist in same vesicles [12, 30], but the existence of separate CGRP and SP vesicles has also been evidenced [17, 31, 32]. Besides, other neuropeptides like neurokinin A can be present together with CGRP and SP [33–35].

The release of CGRP and SP is closely linked to the phenomenon of neurogenic inflammation, which thought to be involved in several painful disorders like migraine, complex regional pain syndrome and sunburn [36–41]. Neurogenic inflammation includes arterial vasodilatation, which is primarily a function of CGRP [42, 43], plasma extravasation from venous vessels and capillaries, which is primarily a function of SP and the neurokinins A and B [44, 45] but can be potentiated by CGRP [46], and possibly mast cell degranulation, which may be species- and organ-specifically induced by CGRP or SP [47–50]. Neurogenic inflammation has been made responsible for mediator cascades ending up in primary and secondary sensitization, which may be closely associated with hyperalgesia and pain [51–53]. Meningeal neurogenic inflammation, particularly plasma extravasation in the cranial dura mater, has been hypothesized to be involved in migraine pain generation, but it is still unclear if this association is merely an experimental phenomenon [41, 54–56]. While vasodilatation of cranial arteries during experimental and spontaneous migraine pain has frequently been described [57–60], although not without contradictory findings [61], a clear proof that meningeal plasma extravasation is present in humans during migraine pain is lacking so far. In line with this discrepancy, there is robust evidence for an increased CGRP release during stimulation of the trigeminal system [62] and during migraine attacks, measured in the venous outflow from the head [63–65] but also in saliva and tear fluid [66–68], whereas early reports about increases in salivary SP levels during migraine are inconsistent [69, 70].

In the present study we set out to measure SP released from the dura mater in our approved hemisected rat head preparation parallel to CGRP release data, part of which has been published before [71]. In this previous publication we have compared the CGRP release between animals pre-treated with fremanezumab, a monoclonal anti-CGRP antibody, with animals pre-treated with a not CGRP binding control antibody. In the present paper we show additional experiments of this kind with correlation of CGRP and SP release, which was boosted by injection of glycerol trinitrate (GTN) to induce orofacial sensitization often associated with migraine [72–75].

## Methods

Animal housing and experiments were carried out according to the German guidelines and regulations of the care and treatment of laboratory animals and the European Communities Council Directive of 24 November 1986 (86/609/EEC), amended 22 September 2010 (2010/63/EU). The experimental protocols were reviewed by an ethics committee and approved by the District Government of Middle Franconia (54-2532.1-21/12).

### Animals

Adult Wistar rats of both sexes (body weight of 12 males: 190–370 g; 6 females: 250–280 g), bred and housed in the animal facility of the Institute of Physiology and Pathophysiology of the Friedrich-Alexander-Universität (FAU) Erlangen-Nürnberg, were kept in a 12-hour light/dark cycle in standard cages in groups of 3–4 and fed with standard food pellets and water ad libitum. The animals were matched and distributed according to their weight, as equally as possible, for the two antibodies used (see below). The oestrus state of the females was not assessed.

### Administration of antibodies

The rats were anaesthetized in a plastic box with an increasing concentration of isoflurane up to 4% (Forene, Abott, Wiesbaden, Germany), applied with an evaporator (Forane Vapor 19.3, Dräger AG, Lübeck, Germany). The animals were weighed, and the neck region was shaved and disinfected with 70% ethanol. Then, 30 mg/kg anti-CGRP antibody, fremanezumab, or isotype control antibody, a human IgG2 antibody-targeting keyhole limpet hemocyanin (Teva Pharmaceuticals, Redwood City, CA, USA) diluted in saline (10 mg/mL) was subcutaneously injected in an even distribution 2 cm left and right from the midline and 5 cm from the caudal of the occiput, using a syringe with a 27-gauge needle. The examiners were blinded as to the identity of the antibodies. The rats were marked at their tail for identification and placed back in their cage, where they recovered from the anaesthesia usually within 2–3 min. The animals were inspected two times on every following day regarding any unusual behaviour.

### Preparation for CGRP and substance P release measurements

Ten or 30 days after the antibody injection, the rats were again shortly anaesthetized by isoflurane to receive an intraperitoneal (i.p.) injection of 5 mg/kg glycerol trinitrate (GTN, 1 mg/mL in saline) using a 23 G needle. Four hours later, the rats were deeply anaesthetized in an atmosphere of an increasing concentration of CO_2_ and killed by bleeding. The head was separated, skinned, and divided in the midline, and the two skull halves with adhering dura mater were prepared for the measurement of the CGRP release according to an approved standard protocol [26, 76]. The skull halves were washed for 30 min with synthetic interstitial fluid (SIF) and mounted in a water bath above warm water (37 °C), holding the temperature constant. The SIF was composed of (in mM): 107.8 NaCl, 3.5 KCl, 0.69 MgSO4 · 7 H_2_O, 26.2 NaHCO3, 1.67 NaH_2_PO4 · 2 H_2_O, 9.64 Na-gluconate, 5.55 glucose, 7.6 sucrose, and 1.53 CaCl_2_ · 2 H_2_O buffered to pH 7.4 with carbogen gas (95% O_2_, 5% CO_2_). The skull halves were filled twice with 500 µL of SIF, followed by a solution of 500 nM capsaicin (dissolved in saline with 1% ethanol and further diluted with SIF) and another SIF; all the applications were at intervals of 5 min. The chosen capsaicin concentration exerts a robust CGRP release [77]. At the end of each interval, the fluid was carefully collected using a pipette without touching the tissue. Two samples of 100 µL each were separated, immediately supplemented with 25 µL of enzyme-immunoassay (EIA) buffer (Bertin Pharma/SPIbio, Montigny le Bretonneux, France) or ELISA buffer (Cayman Chemical, Ann Arbor, MI, USA), respectively, which contain peptidase inhibitors. The samples were immediately deep-frozen and stored at − 20 °C until their analysis within one week.

### Analysis of released CGRP concentration

After thawing, one sample of each experiment was processed with an enzyme immunoassay (EIA) kit for CGRP (Bertin Pharma/SPIbio, Montigny le Bretonneux, France) according to the instructions of the manufacturer. The assay is based on a double-antibody sandwich technique with monoclonal mouse antibodies specific for CGRP (capture antibodies) fixed to the wells of a plastic plate and soluble anti-CGRP tracer antibodies, which recognize another epitope of the CGRP molecule. The tracer antibodies are conjugated with acetylcholine esterase (AchE) that converts Ellman’s reagent to a yellow substance, the absorbance of which is measured at a wavelength of 405 nm using a photo-spectrometer (Opsys MR, Dynex Technologies, Denkendorf, Germany). The intensity of this colour is proportional to the amount of anti-CGRP tracer bound to the CGRP captured in the well and hence proportional to the amount of free CGRP in the samples or in 8 standards containing defined concentrations of CGRP. The final concentration is calculated based on standard curve fitted to the 8 standards. The CGRP assay detects both α- and β-CGRP with a lower limit of 2 pg/mL and has < 0.01% cross-reactivity with other proteins of the calcitonin family. The CGRP concentration in each sample was calculated in pg/mL, considering the added volume of EIA buffer.

### Analysis of released substance P concentration

From the other defrosted sample of each experiment 50 µL were taken for processing with an enzyme-linked immune-assay (ELISA) kit for SP (Cayman Chemical, Ann Arbor, MI, USA) according to the instructions of the manufacturer. The assay is based on the competition between SP in the samples and a conjugate of SP and AchE (tracer, constant amount) for SP-specific rabbit antibodies. These antibodies loaded with SP or the conjugate bind to mouse anti-rabbit IgG (capture antibodies) fixed to the wells. The AchE converts Ellman’s reagent to a yellow substance, which is quantified as described above to be proportional to the amount of tracer bound to the well and hence inversely proportional to the amount of free SP in the samples or in the 8 standards containing defined concentrations of SP. The SP assay has a lower detection limit of 3.9 pg/mL and a cross-reactivity with neurokinin A of 2.7%, according to the manufacturer’s information. The SP concentrations in each sample was calculated in pg/mL, considering the added volume of ELISA buffer.

### Data processing and statistics

For the power calculation we used experiments with fremanezumab treatment as previously reported [71] and calculated post-hoc the effect size (Cohen’s d) for stimulated CGRP release between fremanezumab and control antibody to 1.83. With an α error probability of 0.05 and a 1-β error probability of 0.8, the actual power is 0.98 and the required total sample size is 6, as calculated with G*Power 3.1 (published by the Heinrich Heine Universität Düsseldorf, Germany). Statistical analysis was performed using Statistica software (StatSoft, Tulsa, USA). Analysis of variance (repeated measures ANOVA, one-way ANOVA) extended by Tukey’s honest significant difference (HSD) was applied. Product-moment correlation was used to compare CGRP and SP release data of same experiments. The level of significance was set at *p* < 0.05. Data are displayed as mean ± SEM (standard error of the mean).

## Results

**Fig. 1:**
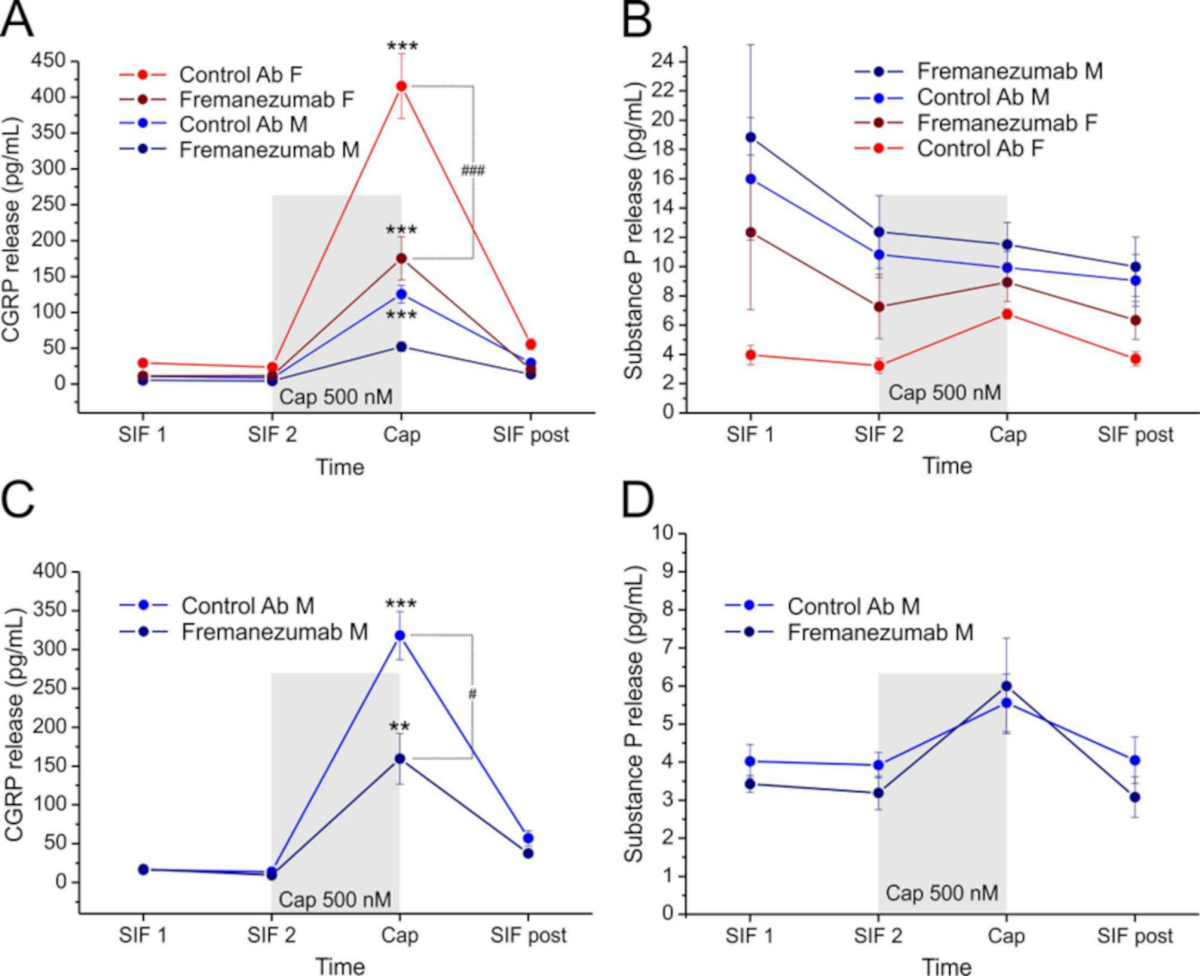
CGRP and SP release from the dura mater in same experiments, 10 days (**A, B**) or 30 days (**C, D**) after injection of fremanezumab or isotype control antibody (Control Ab) in female (F) and male (M) animals. Basal release is determined after periods of 5 and 10 min in buffer (SIF) followed by the release stimulated with 500 nM capsaicin (Cap) and by a post-stimulation period in SIF. Each data point represents the mean ± SEM of 6 experiments. Significant differences between basal (SIF2) and stimulated release (**, *p* < 0.001) and between the two antibodies in stimulated release (##, *p* < 0.001; #, *p* < 0.01)

CGRP and SP release was determined in 24 hemisected cranial preparations from 12 rats (6 male and 6 female animals), half of them pre-treated with fremanezumab and the other half with isotype control antibody 10 days before the final experiments. In the first two samples we determined the basal (unstimulated) release, then capsaicin (500 nM) was applied to provoke stimulated release, and finally a sample without capsaicin was taken as a post-control (Fig. 1A, B). In addition, 12 hemisected preparations from 6 male animals, 3 of them pre-treated with fremanezumab and 3 with control antibody 30 days prior to the final experiments, were treated in the same way (Fig. 1C, D).

### CGRP release

In the experiments of the animals pre-treated with fremanezumab or control antibody 10 days prior to the release experiment, basal and stimulated CGRP release values were analysed using repeated measures ANOVA with the categorical factors, antibody and sex. ANOVA showed significant differences between the repeated measurements (*F*_3,60_ = 172.8, *p* < 0.0001), as expected, but also between antibodies (*F*_1,20_ = 34.8, *p* < 0.0001) as well as sexes (*F*_1,20_ = 52.0, *p* < 0.0001). Therefore, they were differentially displayed in Fig. 1A. The Tukey post-hoc test showed no difference between the two basal values but significant increases in stimulated CGRP release (from the second basal value) in female animals pre-treated with either antibody and in males treated with the control antibody (*p* < 0.001) but not in males treated with fremanezumab (*p* = 0.46). The stimulated values were different between the sexes in fremanezumab treated animals (*p* < 0.01) as well as in control antibody treated animals (*p* < 0.001). A difference in stimulated release values between fremanezumab and control antibody was indicated in female animals (*p* < 0.001) but not in males (*p* = 0.24).

In the additional experiments with male animals treated with antibodies 30 days prior to the release experiment (Fig. 1C), similar differences were seen as in the female animals of the latter groups. ANOVA with repeated measures showed significant differences between the measurements in time (*F*_3,30_ = 88.4, *p* < 0.0001), due to the stimulated release, which was significantly increased both after fremanezumab and control antibody (Tukey test, *p* < 0.001) but also between the antibodies (*p* < 0.01).

### Substance P release

In the experiments of animals pre-treated with fremanezumab or control antibody 10 days prior to the release (Fig. 1B), repeated measures ANOVA with the categorical factors antibody and sex showed a just significant difference between the repeated measurements (*F*_3,60_ = 3.6, *p* < 0.05) and between the sexes (*F*_1,20_ = 14.0, *p* < 0.01) but not between the antibodies (*F*_1,20_ = 3.9, *p* = 0.06). The basal release values seemed to vary but the Tukey post-hoc test showed no difference between specific measurements. Likewise, in the additional experiments with male animals pre-treated 30 days prior to the release (Fig. 1D), no significant differences were detected between the measurements.

### Correlation of CGRP and substance P release

**Fig. 2:**
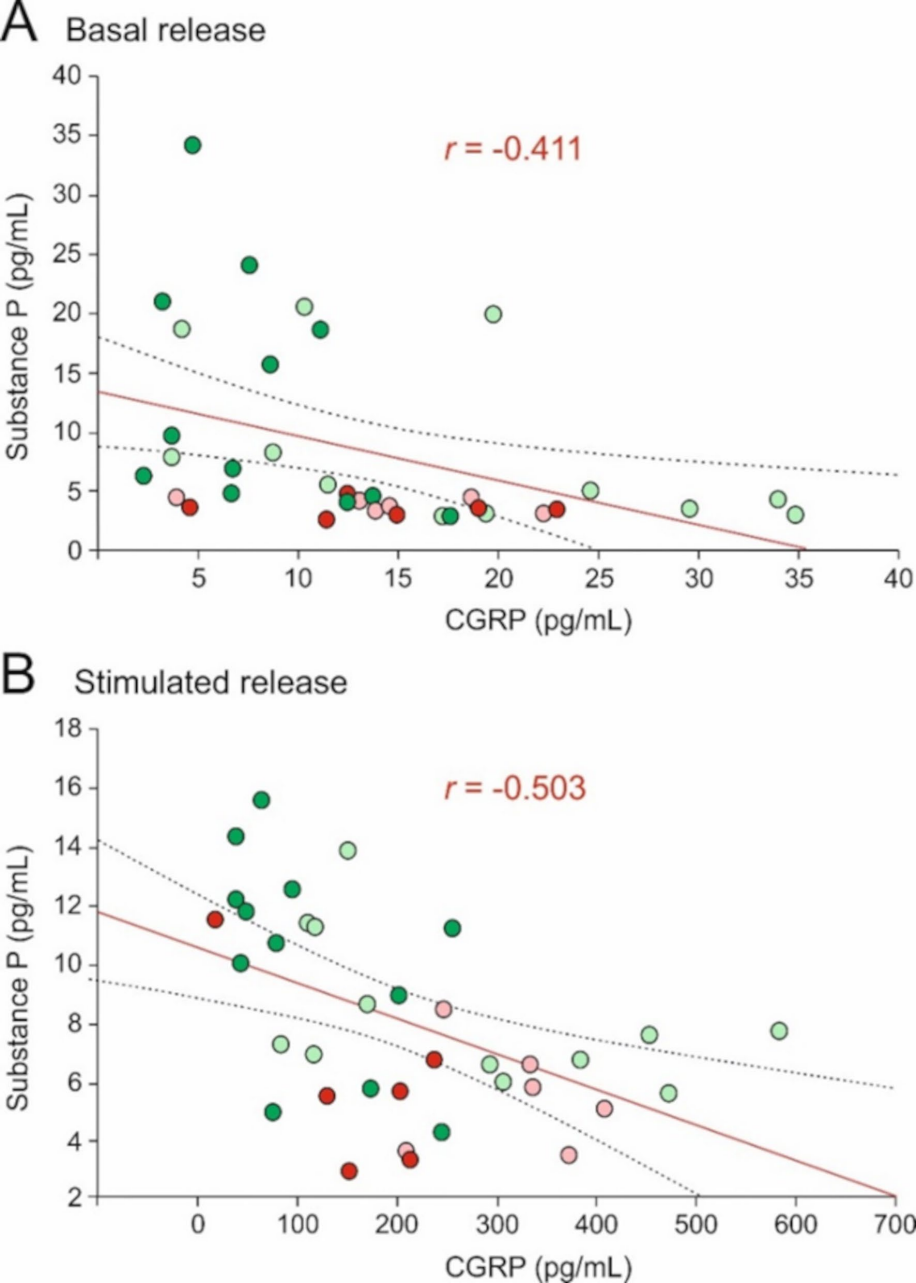
Correlation of CGRP and SP release in same experiments (*n* = 36 hemisected preparations) from rats after 10 days (green circles) or 30 days (red circles) after injection of fremanezumab or isotype control antibody. Dark green/red circles mean data from fremanezumab treated animals, light green/red circles are data from control antibody treated animals. Red line shows regression line, broken lines indicate 95% confidence interval. Significant negative correlation in basal (unstimulated) release (*r* = -0.411) and in capsaicin stimulated release (*r* = -0.503)

The main issue of this study was to compare CGRP and SP measurements in same experiments, independent of sexes and antibody treatment. Overall the experiments indicated that a high CGRP release is associated with a low SP release. To evaluate this, we averaged the two basal values of each experiment and used product-moment correlation to compare basal and stimulated release values of CGRP with respective SP release values. There was a significant negative correlation between the basal release values of CGRP and SP (*N* = 36, *r* = -0.411, *p* < 0.05; Fig. 2A). The significant correlation between the stimulated release values of CGRP and SP was even more negative (*N* = 36, *r* = -0.503, *p* < 0.05; Fig. 2B). Calculating only the 10-day data, the correlation coefficient is -0.459 for the basal release and − 0.520 for the stimulated release values (both significant). Calculating only the 30-day values, the respective coefficient is -0.223 (basal) and − 0.399 (stimulated), both not significant. The non-significant correlation of the 30-day data may partly be due to the lower sample size but also to sex differences. Indeed, when we calculate only male samples, the correlation coefficient is -0.354 for the basal release (not significant) but − 0.613 for the stimulated release values (significant). Calculating only female samples, the correlation coefficient is -0.493 for the basal and − 0.451 for the stimulated release values (both close to significance).

## Discussion

### CGRP and substance P release from rodent dura mater

**Fig. 3:**
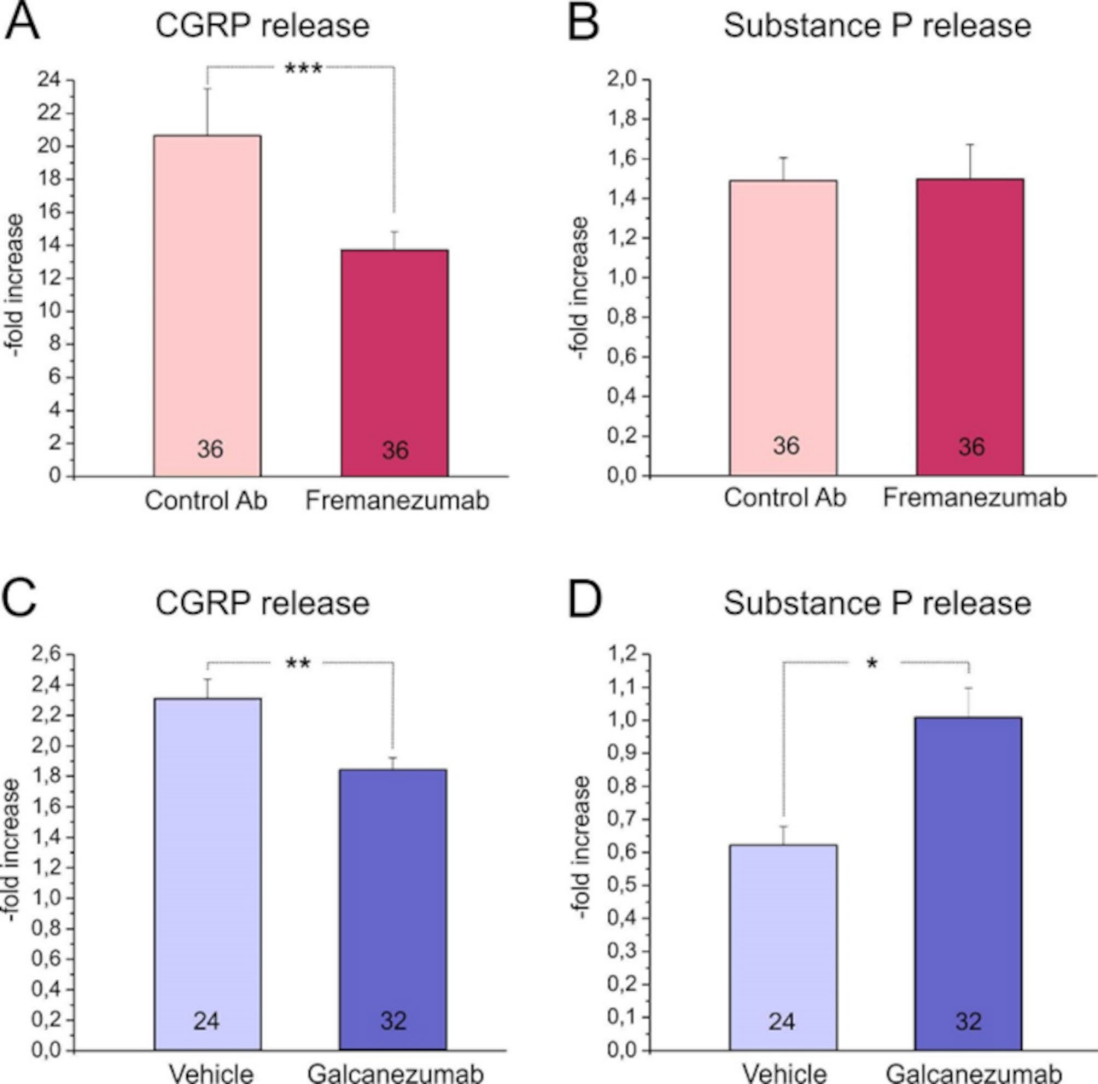
Changes in stimulated CGRP and SP release (-fold of basal release) in experiments with pre-treatment of fremanezumab vs. isotype control antibody (**A, B**) and in experiments with galcanezumab vs. vehicle (**C, D**). Stimulation in **A** and **C** was performed with 500 nM capsaicin, in C and D with 100 nM capsaicin. One-way ANOVA, *** *p* < 0.0005, ** *p* < 0.005, * *p* < 0.05. Numbers in bars represent numbers of experiments. Data are from Dux et al. 2022 and Friedrich et al. 2024

Neuropeptide release from the cephalic dura mater in the hemisected rodent head is a long approved model to test the impact of activating and sensitizing agents on primary meningeal afferents [76–78]. Thereby CGRP release upon electrical or chemical stimulation has been quantified and found to be increased, whereas a significant change in SP release has not been seen [76]. This discrepancy was confirmed in the present study using capsaicin to stimulate primary meningeal afferents. Moreover, in accordance with our previous study [71], we have demonstrated that the anti-CGRP antibody fremanezumab reduces the increase in stimulated CGRP release but did not change SP release. Comparing CGRP and SP release pairwise, the correlation turned even out to be significantly inverse. This is certainly not a specific effect of fremanezumab, since in a recent study of our laboratories another anti-CGRP antibody, galcanezumab, has shown the same differential effect, even associated with an increased SP release compared to control animals not treated with galcanezumab [79]. Thus this effect does also not depend on the treatment of animals with GTN. Figure 3 shows the comparison of data from our previous study measuring CGRP release in animals treated with fremanezumab and with galcanezumab [71, 79]. Thus both fremanezumab and galcanezumab treatment decreases CGRP release and tend to increase SP release. Generally, high CGRP release seems to be associated with low SP release and vice versa.

### Differences in storage and release of CGRP and substance P

The question is, how this discrepancy can evolve. It is generally believed that CGRP and SP are co-localized [80, 81] and even stored in same dense core vesicles and hence co-released upon stimulation of nociceptors [12, 30], although observations of separate vesicles containing either CGRP or SP have also been reported and hence separated release of these peptides has been assumed [1, 12, 17, 31, 32]. Different to our results of a converse correlation of CGRP and SP release from the dura mater, in spinal dorsal horn slices CGRP has been reported to potentiate capsaicin stimulated SP release [82], but this was most likely due to a stimulating effect of CGRP receptors located on separate SP but not CGRP containing central afferent terminals, as it was concluded from more recent patch clamp recordings on medullary slices [83]. The quantity of released CGRP in our study was much higher than that of released SP, which is similarly seen in our previous experiments of this kind [76] but also in similar preparations and other tissues [34, 84]. This is consistent with the frequent observation that in several species more trigeminal and dorsal root ganglion neurons contain CGRP immunoreactivity compared to SP immunoreactivity [80, 85–87] and that nearly all SP immunopositive neurons are also CGRP-positive but not vice versa [88, 89].

### Release mode of CGRP and substance P

The release mode of neuropeptides is not really clear. Stimulated release depends clearly from an increase in intracellular calcium, either by opening of voltage-dependent calcium channels or transient receptor potential (TRP) channels [1, 25, 27–29, 90], but the classical exocytosis as known from synaptic vesicles storing neurotransmitters like glutamate is questionable; at least, typical omega-shaped membrane contacts with dense core vesicles have not been seen [91]. Also, in the dorsal horn of several species, terminals immunoreactive for CGRP and SP have been found to lack immunoreactivity for synaptosome-associated protein of 25 kDa (SNAP-25), which is essential for the classical form of transmitter exocytosis [92], but this observation is partly in conflict with other studies showing that CGRP and SP are associated with synaptosome-like structures [93] and that CGRP release depends on a SNAP-25 complex different to that responsible for the exocytosis of small synaptic vesicles [29].

### Degradation of CGRP and substance P

Notwithstanding these uncertainties and assuming that CGRP and SP are mainly stored in same vesicles, it is reasonable to assume that they are mainly co-released, whatever the nature of the exocytosis mechanisms may be. Hence another mechanism following the release should be taken into account. There is long evidence for an interaction of both neuropeptides regarding their degradation by peptidases [94]. Co-injection of both peptides into the skin has been found to shorten the vasodilatation induced by CGRP, a phenomenon explained by the action of proteases released from mast cells, which are stimulated and degranulated by SP [81]. However, mast cells can also be degranulated by CGRP, which has been explicitly shown to occur in the rodent dura mater [52, 95], so that this could be the result of a reciprocal process of peptide degradation dominated by the peptide with the higher concentration. An imbalance in this process can be expected by the much higher concentrations of released CGRP compared to SP (see Fig. 1), which has been observed in the dura mater as well as in the skin [76, 96].

### Classical preclinical experiments of neurogenic CGRP and substance P effects

Stimulation of meningeal afferents in classical animal experiments has been found leading to plasma protein extravasation from postcapillary vessels in the dura mater [97, 98] and to arterial vasodilatation and increased blood flow, which is mainly a function of CGRP. Neurogenic increases in diameter of rat dural arterial vessels were blocked by the CGRP receptor antagonist CGRP_8 − 37_ but not by the NK1 receptor antagonist RP67580 [46, 99]. Accordingly, electrically evoked increases in meningeal blood flow were significantly reduced by CGRP_8 − 37_ but not by the above mentioned NK1 antagonist [100, 101]. Interestingly, pretreatment of guinea pig basal arteries with SP attenuated slightly the CGRP-induced relaxation [33]. The concentration of SP necessary to cause half-maximal vasodilation of human isolated cerebral arteries was about 100 times higher than that of CGRP [102], whereas in isolated human middle meningeal arteries the vasorelaxing potency of CGRP and substance P was similar [103]. However, in our experiments the stimulated CGRP release was at least 10 times higher than the stimulated substance P release. Taken together, it is conceivable that the low level of substance P released from meningeal afferents does not significantly contribute to vasodilation in the dura mater.

### Clinical relevance

As mentioned already at the beginning, there is inconsistency between CGRP and SP measurements also in clinical studies related to migraine and other primary headaches [68, 69, 104, 105]. While the role of CGRP with its vasodilation potency has long been confirmed by effective migraine therapies using CGRP receptor antagonists [106–108] or monoclonal antibodies targeting CGRP or its receptors [109–111], therapies targeting SP or its main effect, i.e. plasma extravasation, have failed [112]. Thus it is questionable that plasma extravasation has an important role in migraine pathophysiology, although the experimental concept had an important role in understanding neuropeptide mechanisms in migraine [113, 114]. In our present experiments we have applied GTN, bringing the experimental state of the animals closer to the pathophysiology of migraine [72–74]. We have recently shown that the intraperitoneal dose of 5 mg/kg GTN is sufficient to increase CGRP release and cause periorbital hypersensitivity in rats [71, 75] as an expression of facial hyperalgesia, a symptom frequently experienced in migraine. Our results, showing that high CGRP release is associated with low SP release, generate even more doubts about an important role of SP in migraine pathophysiology and question if the experimental phenomenon of plasma protein extravasation exists in migraine at all.

## Data Availability

Data is provided within the manuscript. Raw data and calculations can be provided on demand by the authors.
